# Supplementary material for: Effects of Short-Term Gluten-Free Diet on Cardiovascular Biomarkers and Quality of Life in Healthy Individuals: A Prospective Interventional Study
Source: Nutrients. 2024 Jul 13;16(14):2265. doi: 10.3390/nu16142265 (PMC11279490; doi:10.3390/nu16142265)
Supplement: Supplementary file 1 [file nutrients-16-02265-s001.zip › nutrients-3096175-supplementary.pdf]

Supplement Table S1: Results of the Olink® plasma proteomics, inflammation panel (95302).

Assay	UniProt ID	NPX difference	P-value	Assay	UniProt ID	NPX difference	P-value
SIRT2	Q8IXJ6	-0.5022	0.1001	FGF-5	P12034	-0.0502	0.6288
CD40	P25942	-0.1662	0.121	MCP-4	Q99616	-0.1018	0.6355
CCL3	P10147	-0.2972	0.1278	TGF-alpha	P01135	-0.0446	0.6404
STAMBP	O95630	-0.2671	0.1369	IL-24	Q13007	0.1601	0.6516
IL10	P22301	-0.2394	0.1446	CDCP1	Q9H5V8	-0.0803	0.6672
4E-BP1	Q13541	-0.5606	0.1505	IL4	P05112	-0.2466	0.671
MCP-1	P13500	-0.2308	0.1608	OPG	O00300	-0.0759	0.6739
TRANCE	O14788	-0.2523	0.1837	CCL25	O15444	-0.1004	0.6799
CXCL9	Q07325	-0.3646	0.1904	IL-10RA	Q13651	-0.0578	0.6995
IL18	Q14116	-0.2731	0.2229	CD5	P06127	-0.0675	0.7038
IL8	P10145	-0.2635	0.2426	IL-12B	P29460	-0.1144	0.7045
IL2	P60568	0.1117	0.2482	LIF	P15018	-0.0368	0.7199
CCL4	P13236	-0.2161	0.2555	LIF-R	P42702	-0.0304	0.7582
IL6	P05231	-0.2435	0.2611	CCL19	Q99731	-0.0709	0.7628
MCP-3	P80098	-0.1585	0.2859	SLAMF1	Q13291	0.0495	0.7635
EN-RAGE	P80511	-0.2142	0.2919	TWEAK	O43508	-0.0511	0.7641
TRAIL	P50591	-0.1391	0.3	IL-10RB	Q08334	-0.0483	0.7698
AXIN1	O15169	-0.2845	0.3025	IL33	O95760	-0.069	0.7744
IL-20	Q9NYY1	-0.0782	0.3088	TSLP	Q969D9	0.1272	0.7804
CCL20	P78556	0.3775	0.3532	CXCL11	O14625	-0.0777	0.7816
CXCL10	P02778	-0.2167	0.3535	IL7	P13232	-0.0715	0.7967
IL-22 RA1	Q8N6P7	-0.3606	0.3646	CCL28	Q9NRJ3	-0.0504	0.8002
CSF-1	P09603	-0.0777	0.38	LAP TGF-beta-1	P01137	-0.042	0.8038
CX3CL1	P78423	-0.1258	0.3869	Flt3L	P49771	-0.0443	0.8071
VEGFA	P15692	-0.1022	0.3944	FGF-23	Q9GZV9	0.1379	0.8199
IFN-gamma	P01579	-0.3289	0.4223	NRTN	Q99748	0.0384	0.8295
CASP-8	Q14790	-0.1251	0.4254	CCL11	P51671	-0.0299	0.8337
IL5	P05113	0.1543	0.4311	CXCL6	P80162	-0.0875	0.8353
IL13	P35225	0.2398	0.4603	IL-15RA	Q13261	-0.0286	0.8501
DNER	Q8NFT8	-0.0607	0.4708	FGF-19	O95750	0.0681	0.8514
PD-L1	Q9NZQ7	-0.1186	0.4987	ARTN	Q5T4W7	-0.0565	0.8627
HGF	P14210	-0.1065	0.5048	SCF	P21583	0.0308	0.8678
IL-1 alpha	P01583	0.0875	0.5057	CST5	P28325	-0.0324	0.8725
TNFSF14	O43557	-0.1349	0.5252	CCL23	P55773	-0.0338	0.8796
ADA	P00813	-0.1307	0.5389	TNFB	P01374	-0.0286	0.9
uPA	P00749	-0.1044	0.5394	IL-17A	Q16552	-0.0304	0.9278
TNF	P01375	-0.1281	0.5456	CXCL1	P09341	-0.0441	0.929
GDNF	P39905	-0.1072	0.5486	CD6	P30203	-0.0117	0.9469
MMP-10	P09238	-0.1312	0.5687	CXCL5	P42830	0.0519	0.95
IL-18R1	Q13478	-0.0791	0.5766	FGF-21	Q9NSA1	-0.0379	0.9556
ST1A1	P50225	-0.2032	0.5906	CD8A	P01732	-0.0141	0.9637
OSM	P13725	-0.1757	0.5953	MMP-1	P03956	-0.0183	0.9654
IL-2RB	P14784	-0.1416	0.6037	NT-3	P20783	-0.0045	0.9667
CD244	Q9BZW8	-0.0621	0.6157	MCP-2	P80075	-0.0116	0.9672
TNFRSF9	Q07011	-0.0968	0.6162	Beta-NGF	P01138	0.0008	0.9802
IL-17C	Q9P0M4	-0.1218	0.6187	IL-20RA	Q9UHF4	-0.004	0.9873

Supplement Table S2: Results of the Olink® plasma proteomics, cardiovascular II panel (95500).

Assay	UniProt ID	NPX difference	P-value	Assay	UniProt ID	NPX difference	P-value
SRC	P12931	-0.7681	0.0241	SERPINA12	Q8IW75	-0.3121	0.6821
HSP 27	P04792	-0.4793	0.0673	TNFRSF10A	O00220	-0.0747	0.6828
STK4	Q13043	-0.5312	0.1021	THBS2	P35442	0.0424	0.6852
GT	P51161	-0.3627	0.1419	SLAMF7	Q9NQ25	0.0666	0.6887
CCL3	P10147	-0.2329	0.2085	IgG Fc receptor II-b	P31994	0.0623	0.692
MMP7	P09237	-0.2069	0.232	Dkk-1	O94907	0.1117	0.706
DECR1	Q16698	-0.5002	0.233	BMP-6	P22004	0.0442	0.7217
GLO1	Q04760	-0.3348	0.2429	hOSCAR	Q8IYS5	0.0318	0.7382
KIM1	Q96D42	-0.2573	0.316	NEMO	Q9Y6K9	-0.1025	0.745
HO-1	P09601	-0.1623	0.3192	PTX3	P26022	0.0618	0.7491
SOD2	P04179	0.0502	0.3398	LEP	P41159	0.2311	0.7523
FABP2	P12104	-0.2858	0.3587	SORT1	Q99523	0.0519	0.7604
PGF	P49763	-0.1451	0.3593	CA5A	P35218	-0.1188	0.766
IL18	Q14116	-0.2061	0.3601	IL-17D	Q8TAD2	-0.0317	0.7717
TGM2	P21980	-0.2637	0.3765	PD-L2	Q9BQ51	0.0389	0.7754
TF	P13726	-0.1288	0.3772	PAPPA	Q13219	0.0473	0.7963
ANGPT1	Q15389	0.5058	0.3934	PRSS8	Q16651	0.0408	0.8026
FS	P19883	-0.1396	0.4001	ADAM-TS13	Q76LX8	0.0187	0.812
PDGF subunit B	P01127	0.6056	0.4273	AMBP	P02760	0.0171	0.8157
PARP-1	P09874	0.1506	0.4296	REN	P00797	-0.0435	0.8228
GDF-2	Q9UK05	-0.1644	0.437	CTRC	Q99895	-0.0526	0.8288
BNP	P16860	0.3088	0.4426	IL1RL2	Q9HB29	-0.0519	0.8541
CD84	Q9UIB8	0.1076	0.4777	IL16	Q14005	0.0511	0.8643
IL-27	Q8NEV9,Q14213	-0.1556	0.4963	LOX-1	P78380	-0.0562	0.8756
CD40-L	P29965	0.2968	0.502	DCN	P07585	-0.011	0.9039
SPON2	Q9BUD6	-0.0596	0.5208	FGF-21	Q9NSA1	-0.081	0.9089
ADM	P35318	-0.0948	0.5387	IL-1ra	P18510	-0.0197	0.909
MERTK	Q12866	-0.1149	0.5517	IDUA	P35475	-0.0234	0.9091
IL-4RA	P24394	0.0736	0.5839	MARCO	Q9UEW3	0.01	0.9191
TNFRSF11A	Q9Y6Q6	-0.0822	0.5967	TNFRSF13B	O14836	0.0158	0.9222
CTSL1	P07711	-0.0787	0.6077	LPL	P06858	0.0127	0.9291
ITGB1BP2	Q9UKP3	-0.089	0.6145	TM	P07204	0.0134	0.9335
PAR-1	P25116	-0.0813	0.6146	SCF	P21583	-0.0135	0.9367
IL6	P05231	-0.0844	0.6175	HAOX1	Q9UJM8	0.0653	0.9387
CCL17	Q92583	0.1692	0.6285	PSGL-1	Q14242	0.009	0.9421
AGRP	O00253	-0.087	0.6346	THPO	P40225	-0.0095	0.9431
GH	P01241	-0.4944	0.6363	CD4	P01730	-0.0079	0.952
VSIG2	Q96IQ7	0.0748	0.6451	PRELP	P51888	-0.005	0.9577
ACE2	Q9BYF1	-0.0664	0.646	VEGFD	O43915	0.0062	0.9604
BOC	Q9BWV1	0.0554	0.6538	RAGE	Q15109	0.0069	0.9647
HB-EGF	Q99075	0.1266	0.6589	TIE2	Q02763	0.0059	0.9662
PIgR	P01833	0.0194	0.6636	CXCL1	P09341	-0.0196	0.9673
FGF-23	Q9GZV9	0.2258	0.6638	PRSS27	Q9BQR3	-0.0077	0.9689
Gal-9	O00182	-0.0558	0.6654	GIF	P27352	-0.0121	0.9703
MMP12	P39900	-0.101	0.6739	TRAIL-R2	O14763	-0.0033	0.9806
XCL1	P47992	-0.0932	0.6757	CEACAM8	P31997	0.0005	0.9989
